# Association of *helicobacter pylori* infection and chronic atrophic gastritis with risk of colonic, pancreatic and gastric cancer: A ten-year follow-up of the ESTHER cohort study

**DOI:** 10.18632/oncotarget.7946

**Published:** 2016-03-06

**Authors:** Xin-Zu Chen, Ben Schöttker, Felipe Andres Castro, Hongda Chen, Yan Zhang, Bernd Holleczek, Hermann Brenner

**Affiliations:** ^1^ Department of Gastrointestinal Surgery, West China Hospital, Sichuan University, Chengdu, China; ^2^ Division of Clinical Epidemiology and Aging Research, German Cancer Research Center (DKFZ), Heidelberg, Germany; ^3^ Saarland Cancer Registry, Saarbrücken, Germany; ^4^ German Cancer Consortium (DKTK), Heidelberg, Germany

**Keywords:** helicobacter pylori, chronic atrophic gastritis, colon cancer, pancreatic cancer, gastric cancer

## Abstract

**Objectives:**

To assess the association of *H. pylori* and chronic atrophic gastritis (AG) with colonic, pancreatic and gastric cancer in a population-based prospective cohort.

**Methods:**

Serum antibodies against *H. pylori* in general and specific to cytotoxin-associated gene A (CagA), as well as serum pepsinogen I and II were analyzed in 9,506 men and women, aged 50–75 years in a cohort study from Saarland, Germany. Incident cases of colonic, pancreatic and gastric cancer were ascertained by record linkage with data from the Saarland Cancer Registry.

**Results:**

During an average follow-up of 10.6 years, 108 colonic, 46 pancreatic and 27 gastric incident cancers were recorded. There was no association between *H. pylori* infection and colonic cancer (HR = 1.07; 95% CI 0.73–1.56) or pancreatic cancer (HR = 1.32; 0.73–2.39), regardless of either CagA seropositivity or AG status. In contrast, CagA+ infection was associated with a strongly increased risk of gastric cancer, especially non-cardia gastric cancer, and this association was particularly pronounced in the presence of AG. Compared to people without AG and without CagA+ infection, people with both risk factors had a significantly increased risk of non-cardia gastric cancer (HR = 32.4; 7.6–137.6).

**Conclusions:**

This large cohort study did not observe an association of *H. pylori* infection or AG with colonic or pancreatic cancer, but underlines that the vast majority of non-cardia gastric cancers arise from AG and infection with CagA+ *H. pylori* strains.

## INTRODUCTION

*Helicobacter pylori* (*H. pylori*) is well-known as a carcinogenic pathogen for gastric cancer [1]. Moreover, *H. pylori* infection has been suggested to be associated with other cancers of the digestive tract including colonic and pancreatic cancer [2–4], but results were inconclusive. [5, 6] To our knowledge, no previous cohort study has simultaneously assessed the risk of various gastrointestinal cancers in the same study population.

Furthermore, *H. pylori* infection shows a strong association with chronic atrophic gastritis (AG), which is considered as a precancerous lesion of the gastric mucosa [7]. *H. pylori* infection is likely to be primarily involved in the early stage of development of AG, and less so in the development of gastric cancer from AG [8]. In fact, clearance of the *H. pylori* infection may often occur in advanced stages of AG [9].

The 4th European Helicobacter Study Group's Maastricht IV/Florence Consensus Report stated that the combination of *H. pylori* infection and AG determined by serological examination is suiTable for the identification of subjects with a high risk of gastric cancer [10]. The European Helicobacter Study Group recommends that differences in *H. pylori* virulence factors shall be taken into account to identify subjects with high risk of gastric cancer because the oncogenic potential of *H. pylori* infections strongly varies according to the presence of virulent strains [10]. For example, the cytotoxin-associated gene A (CagA) strain is thought to contribute to cancer development by steps from inflammation to atrophy and then cancer [11–13].

However, data are scarce on the joint prediction of gastric cancer risk by both *H. pylori* infection and presence of AG while taking major virulence factors into account. Such data are crucial to estimate the potential of serological screen and treat strategies ahead of their implementation. In this article, we report such results from a large population-based cohort study from Germany, paying attention not only to gastric cancer but also to colonic and pancreatic cancer.

## RESULTS

The prevalences of overall (IgG+/anyCagA), CagA– and CagA+ *H. pylori* infection at baseline were 49.9%, 22.4%, and 27.5%, respectively. Compared with non-infected participants, *H. pylori* infected participants were older (*p* < 0.001), had lower education levels (*p* < 0.001) and reported to consume less tobacco (*p* = 0.030) and alcohol (*p* < 0.001) (Table 1). Among *H. pylori* infected persons, those with CagA+ strains included slightly higher proportions of females (*p* = 0.019) and never smokers (*p* = 0.002) than those with CagA–strains.

**Table 1:** Characteristics of population by H. *pylori* infectionand CagA seropositivityat baseline

Characteristics	Total	Non-infected		*H. pylori* infected		*P* value †	*P* value ‡
		IgG−/CagA−	IgG+/any CagA	IgG+/CagA−	IgG+/CagA+		
		*n* (%)	*n* (%)	*n* (%)	*n* (%)		
Observations (*n*)	9,506	4,767	4,739	2,128	2,611		
Prevalence		—	49.9%	22.4%	27.5%		
Age (years)						**< 0.001**	0.562
50–54	1,627	925 (19.4)	702 (14.8)	322 (15.1)	380 (14.6)		
55–59	1,616	922 (19.3)	694 (14.6)	321 (15.1)	373 (14.3)		
60–64	2,590	1,285 (27.0)	1,305 (27.5)	580 (27.3)	725 (27.8)		
65–69	2,176	998 (20.9)	1,178 (24.9)	512 (24.1)	666 (25.5)		
70–75	1,497	637 (13.4)	860 (18.2)	393 (18.5)	467 (17.9)		
Sex						0.438	**0.019**
Female	5,215	2,634 (55.3)	2,581 (54.5)	1,119 (52.6)	1,462 (56.0)		
Male	4,291	2,133 (44.7)	2,158 (45.5)	1,009 (47.4)	1,149 (44.0)		
Education (years)						**< 0.001**	0.703
≤ 9	6,933	3,339 (71.5)	3,594 (78.2)	1,620 (78.4)	1,974 (78.1)		
10–11	1,302	738 (15.8)	564 (12.3)	259 (12.5)	305 (12.1)		
≥ 12	1,030	594 (12.7)	436 (9.5)	187 (9.1)	249 (9.9)		
Smoking status						**0.030**	**0.002**
Never	4,623	2,277 (48.9)	2,346 (51.2)	1,004 (48.5)	1,342 (53.3)		
Former	3,049	1,567 (33.6)	1,482 (32.3)	705 (34.1)	777 (30.9)		
Current	1,571	814 (17.5)	757 (16.5)	359 (17.4)	398 (15.8)		
Alcohol consumption						**< 0.001**	0.187
Abstainer	2,792	1,315 (30.2)	1,477 (34.9)	644 (33.7)	833 (35.8)		
Women 0–19.99 g/d or Men 0–39.99 g/d	5,200	2,697 (62.0)	2,503 (59.1)	1,150 (60.2)	1,353 (58.2)		
Women ≥ 20 g/d or Men ≥ 40 g/d	591	338 (7.8)	253 (6.0)	115 (6.0)	138 (5.9)		

Abbreviations: CagA, cytotoxin-associated gene A; IgG, immunoglobulin G; *H. pylori*, *Helicobacter pylori*.

† *P* value, for comparison between non-infected (IgG–/CagA–) participants and all *H. pylori* infected (IgG+/any CagA) participants.

‡ *P* value, for comparison between participants with IgG+/CagA+ and IgG+/CagA− *H. pylori* infection.

**Figure 1:**
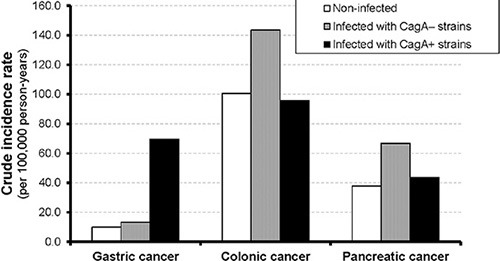
Crude incidence rate (per 100,000 person-years) of gastric, colonic and pancreatic cancer by H. *pylori* infection and CagA serostatus at baseline

During a median follow-up of 10.6 years, 27 gastric, 108 colonic and 46 pancreatic incident cancers were recorded, respectively (Table 2). Crude incidence rates were 26.97, 108.94 and 45.96 per 100,000 person-years for gastric, colonic and pancreatic cancer, respectively. Participants with *H. pylori* infection had much higher incidence of gastric cancer than non-infected participants, whereas no such pattern was seen for colonic or pancreatic cancer (Figure 1). Furthermore, gastric cancer incidence was much higher among participants infected with CagA+ *H. pylori* strains than among those infected with CagA– *H. pylori* strains. Nevertheless, even among those with CagA+ *H. pylori* infection, gastric cancer incidence was still lower than colonic cancer incidence.

**Table 2:** Incidence and risk of gastric, colonic and pancreatic cancer by H. *pylori* infection and CagA seropositivity

Cancers	Total	Non-infected		*H. pylori* infected	
		IgG−/CagA−	IgG+/any CagA	IgG+/CagA−	IgG+/CagA+
**Gastric cancer (observations, *n*)**	9,497	4,760	4,737	2,127	2,610
Cases (*n*)	27	5	22	3	19
Crude incidence rate†	26.97	9.96	44.08	13.32	69.37
HR_1_ (95% CI)‡	—	Ref	**3.91 (1.48–10.38)**	1.16 (0.28–4.86)	**6.27 (2.33–16.86)**
HR_2_ (95% CI)‡	—	—	—	Ref	**5.57 (1.64–18.89)**
**Colonic cancer (observations, *n*)**	9,443	4,733	4,710	2,120	2,590
Cases (*n*)	108	50	58	32	26
Crude incidence rate†	108.94	100.61	117.32	143.55	95.78
HR_1_ (95% CI)‡	—	Ref	1.07 (0.73–1.56)	1.30 (0.83–2.03)	0.87 (0.54–1.14)
HR_2_ (95% CI)‡	—	—	—	Ref	0.68 (0.40–1.14)
**Pancreatic cancer (observations, *n*)**	9,504	4,766	4,738	2,128	2,610
Cases (*n*)	46	19	27	15	12
Crude incidence rate†	45.96	37.86	54.11	66.78	43.74
HR_1_ (95% CI)‡	—	Ref	1.32 (0.73–2.39)	1.61 (0.82–3.18)	1.08 (0.52–2.24)
HR_2_ (95% CI)‡	—	—	—	Ref	0.67 (0.31–1.43)

Abbreviations: CagA, cytotoxin-associated gene A; CI, confidence interval; IgG, immunoglobulin G; *H. pylori*, *Helicobacter pylori*; HR, hazard ratio.

† Per 100,000 person-years.

‡ Adjusted for age, sex, education level, smoking status and alcohol consumption. HR_1_, comparing infected (IgG+) and non-infected (IgG–/CagA–) participants. HR_2_, comparing participants with IgG+/CagA+ and IgG+/CagA– *H. pylori* infection.

**Figure 2:**
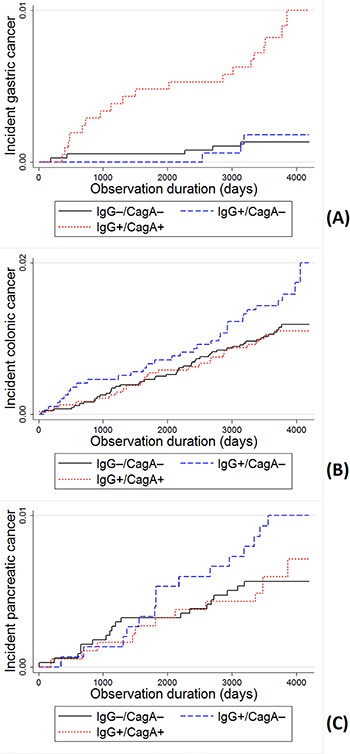
Incidence of cancer by H. *pylori* IgG and CagA serostatus Kaplan-Meier curves and Log-rank test of the incidence of (**A**) gastric, (**B**) colonic and (**C**) pancreatic cancer. Significant differences were observed for gastric cancer incidence in comparisons of virulent strains to non-infected (*p* < 0.0001), and virulent strains to non-virulent strains (*p* = 0.003), but not in non-virulent strains to non-infected (*p* = 0.686). No significant differences were observed for colonic and pancreatic cancer incidence (*p* > 0.05).

In Cox regression models, very strong associations of *H. pylori* infection were found with gastric cancer (adjusted hazard ratios [95% CIs] for any and CagA+ *H. pylori* infection: 3.91 [1.48–10.38] and 6.27 [2.33–16.86], respectively), but not with the other gastrointestinal cancers (Table 2, Figure 2).

As shown in Table 3, serological evidence of AG was associated with an increased risk of gastric cancer (HR = 3.45, 95% CI 1.38–8.64), and CagA+ *H. pylori* infection was associated with a strongly increased risk of gastric cancer regardless of serological evidence of AG.

**Table 3:** Risk of gastric cancer incidence by chronic atrophic gastritis, H. *pylori* infection and CagA seropositivity

Chronic atrophic gastritis	*H. pylori* infection	Sample (*n*)	Cases (*n*)	Crude incidence rate†	HR_1_ (95% CI)‡	HR_2_ (95% CI)‡
None	IgG−/CagA−	4,555	5	10.42	Ref	Ref
	IgG+/CagA−	1,984	2	9.54		0.79 (0.15–4.08)
	IgG+/CagA+	2,295	14	58.13		**5.05 (1.81–14.09)**
Present	IgG−/CagA−	205	0	0	**3.45 (1.38–8.64)**	Not estimable
	IgG+/CagA−	143	1	64.61		5.50 (0.63–47.57)
	IgG+/CagA+	315	5	151.20		**12.51 (3.57–43.22)**

Abbreviations: CagA, cytotoxin-associated gene A; CI, confidence interval; *H. pylori*, *Helicobacter pylori*; HR, hazard ratio.

† Per 100,000 person-years.

‡ Adjusted for age, sex, education level, smoking status and alcohol consumption. HR_1_, comparing participants with and without chronic atrophic gastritis. HR_2_, comparing different groups with participants without chronic atrophic gastritis and without *H. pylori* infection.

Even stronger associations were observed when the analyses were restricted to non-cardia gastric cancer, and the risk was particularly high in the presence of both AG and CagA+ *H. pylori* infection (Table 4). Compared to people without AG and without CagA positive infection, people with both risk factors had a 32.4-fold risk of non-cardia gastric cancer (95% CI 7.6–137.6).

**Table 4:** Risk of developing non-cardia gastric cancer by chronic atrophic gastritis, H. *pylori* infection and CagA seropositivity

Chronic atrophic gastritis	*H. pylori* infection	Sample (*n*)	Cases (*n*)	Crude incidence rate†	HR (95% CI)‡
Any	IgG−/CagA−, or IgG+/CagA−	6,883	4	5.50	Ref
Any	IgG+/CagA+	2,608	17	62.09	**18.06 (2.42–134.92)**
None	Any	8,828	15	16.13	Ref
Present	Any	663	6	84.73	**4.77 (1.82–12.49)**
None	IgG−/CagA−, or IgG+/CagA−	6,535	3	4.35	Ref
Present	IgG−/CagA−, or IgG+/CagA−	348	1	26.49	5.55 (0.57–53.97)
None	IgG+/CagA+	2,293	12	49.85	**11.21 (3.16–39.84)**
Present	IgG+/CagA+	315	5	151.20	**32.39 (7.63–137.62)**

Abbreviations: CagA, cytotoxin-associated gene A; CI, confidence interval; *H. pylori*, *Helicobacter pylori*; HR, hazard ratio.

† Per 100,000 person-years

‡ Adjusted for age, sex, education level, smoking status and alcohol consumption.

No association between AG and risk of colonic or pancreatic cancer was found (Table 5).

**Table 5:** Association between chronic atrophic gastritis and colonic or pancreatic cancer

Cancer	Chronic atrophic gastritis†	Sample (*n*)	Cases (*n*)	Crude incidence rate‡	HR (95% CI)§
Colonic cancer	None	8,777	99	107.58	Ref
	Mild-moderate	305	5	153.88	1.29 (0.52–3.18)
	Severe	361	4	103.52	0.81 (0.30–2.20)
Pancreatic cancer	None	8,835	42	45.20	Ref
	Mild-moderate	307	2	60.89	1.32 (0.32–5.49)
	Severe	362	2	51.63	1.09 (0.26–4.52)

Abbreviations: CI, confidence interval; HR, hazard ratio.

† Mild-moderate and severe AG are defined as serostatus: (i) 20 ng/mL ≤ pepsinogen (PG) I < 70 ng/mL and PG I/II ratio < 3.0, and (ii) PG < 20 ng/mL and PG I/II ratio < 3.0), respectively [48].

‡ Per 100,000 person-years.

§ Adjusted for age, sex, education level, smoking status and alcohol consumption.

## DISCUSSION

To our knowledge, this is the first study that simultaneously assessed the associations of *H. pylori* infection and AG with gastric, colonic and pancreatic cancer in the same study population. We did not find any association of *H. pylori* infection or AG with colonic or pancreatic cancer. In contrast, very strong associations were found with gastric cancer, especially non-cardia gastric cancer. Participants with serological evidence of both AG and CagA+ *H. pylori* infection had a 32-fold risk of non-cardia gastric cancer compared to participants with neither of these conditions.

Our study confirms the strong association between *H. pylori* infection and gastric cancer development reported in previous cohort studies from high incidence countries with high prevalence of *H. pylori* infection, such as Japan, Korea and China [13–16]. AmongWestern populations, previous observational studies also identified *H. pylori* as a risk factor for gastric cancer, but few had a prospective cohort design [17–19]. In a meta-analysis of epidemiological studies published until June 2009, it remained unclear whether additional stratification by virulent *H. pylori* strains has an additional benefit in identification of those with the highest risk for gastric cancer [20]. Our study agrees with another large prospective cohort study published after the review that risk stratification by virulence factors is indeed important [21]. In our study, we found that participants infected with non-virulent strains (CagA–) did not have an increased risk of developing gastric cancer, whereas those infected with virulent strains (CagA+) did. Among many *H. pylori* specific proteins, it is widely acknowledged that CagA, generated from virulent strains, induces extensive inflammation and progression to more severe disease states [22]. Therefore, if *H. pylori* testing is carried out, the serological CagA status should also be determined to better judge the gastric cancer risk. Furthermore, previous studies showed that *H. pylori* infection might be related to gastric cancer through its influence on the onset and progression of AG [13, 23]. A previous longitudinal analysis of the ESTHER study showed that infection with *H. pylori* was a strong predictor for the development of AG [24]. In particular, study participants with infection of virulent strains (CagA+) had a strongly increased incidence of AG. The current study extends this finding by showing a dramatically increased risk of non-cardia gastric cancer in the presence of both AG and CagA+ *H. pylori* infection.

In the light of these results from epidemiological studies, *H. pylori* eradication is highly likely and effective approach for gastric cancer prevention. However, the evidence from randomized controlled trials (RCTs) was very sparse until the so far largest study recently published updated results with 15-year follow-up data [25, 26]. The result of this Chinese study has recently been meta-analyzed with the five other RCTs conducted in middle and late adulthood so far [27]. In summary, gastric cancer risk was statistically significantly reduced by 36% by *H. pylori* eradication. Given that *H. pylori* is mostly acquired in childhood even stronger reduction of gastric cancer risk might be achieved by eradication earlier in life, but this will be difficult to demonstrate by clinical trials which would take decades to complete.

Our study underlines that the vast majority of gastric cancers arise from AG and infection with CagA+ *H. pylori* strains. These two high-risk groups should undergo different further treatment. The European Helicobacter Study Group agreed on the following statement “Serological screening is suitable for clinical use in countries with a relatively low incidence of gastric cancer, because it enables endoscopic follow-up of cases with an abnormal serological profile suggesting atrophic gastritis” [10, 28]. Endoscopy enables the detection and resection of early malignancies and has a high potential to prevent gastric cancer mortality in AG patients [29]. Eradication treatment of *H. pylori* infection among patients with any premalignant lesions is recommended by all major international guidelines [30]. Our study results imply that additionally, all participants free of AG but with CagA positive *H. pylori* infection are high-risk patients and should receive eradication treatment. A cost-effectiveness study estimated that screening for and treatment of CagA+ *H. pylori* strains has a similar cost-effectiveness compared to screening for and treating of all *H. pylori* infected individuals in the United States, Finland, Columbia, Japan and countries with similar *H. pylori* prevalence and gastric cancer incidence [31]. The limitations of the cited study were that it did not include screening for AG and did not consider potential negative effects by antibiotic treatment. Cost-effectiveness of a combined serological screening and treatment study for AG and CagA+ *H. pylori* infection will likely be higher because more premalignant cancers would be detected among AG patients and fewer individuals with low gastric cancer risk (*H. pylori*+/CagA– individuals) would receive antibiotic treatment reducing the risk for adverse events and resistant bacteria. However, currently no organized screening programs exist outside the high-risk countries Japan and South Korea [32].

Colonic cancer has also been linked to *H. pylori* infection by previous studies. A recent meta-analysis summarized 17 case-control studies for the outcome colon cancer and 16 for the outcome colon polyps and obtained modest but statistically significant associations of *H. pylori* infection with both outcomes [33]. However, only three studies had a prospective design and all three studies did not observe an association of *H. pylori* infection with colonic canceror colon polyps [34–36]. Our prospective cohort study does support the findings of the previous prospective studies and extends their results by adding that stratification by CagA seropositivity or AG also did not lead to the observation of an association of *H. pylori* infection with colon cancer incidence.

Likewise, inconsistent results for an association between *H. pylori* infection and pancreatic cancer have been reported although the recent review of Xiao et al. [6]. did not observe heterogeneity across 9 included studies and reporting strong effect estimates from meta-analysis. However, comparing the 3 prospective nested case-control studies reveals that only one study from Finland observed a significant association between *H. pylori* infection and CagA+ with pancreatic cancer, whereas other studies from Sweden and the United states did not observe this association [37–39]. Our study results are in agreement with the latter studies that do not support a major etiological role of *H. pylori* in pancreatic cancer development. Interestingly, a recent meta-analysis based on five prospective nested case-controlstudies found the infection with CagA-negative non-virulent *H. pylori* might be associated with increased risk of pancreatic cancer development [40]. In summary, current study results about the association of *H. pylori* infection and pancreatic cancer are conflicting and further prospective, ideally larger studies are required to assess whether *H. pylori* infection is associated with pancreatic cancer.

There are some limitations of our study that should be kept in mind when interpreting our results. First, in our study with 10 years of follow-up, only 27 incident gastric cancers occurred, which precluded more detailed analysis, e.g. by sex or major age groups. However, the associations of AG and CagA+ *H. pylori* infection with gastric cancer incidence were so strong that they were highly statistically significant despite the small case number. Nevertheless, the confidence intervals are wide and the strength of the observed associations could not be estimated with high precision. Second, we did not assess other important virulent factors, such as vacuolating cytotoxin A (VacA), *helicobacter* cysteine-rich protein C (HcpC), and chaperonin (GroEL), which might be informative in addition to CagA. Those virulent proteins of *H. pylori* were previously found to be potential risk factors associated with AG, gastric cancer, or colonic cancer [41–43]. Finally, information on eradication treatment for *H. pylori* infection after baseline was not available, which could have influenced our findings.

In summary, we did not observe an association between *H. pylori* infection as well as AG with colonic or pancreatic cancer in this population-based prospective study which does not support suggestions from previous case-control studies that *H. pylori* infection increases extragastric malignancy risk. On the contrary, our study underlines that the vast majority of non-cardia gastric cancers arise from AG and infection with CagA+ *H. pylori* strains. These study results underline that subjects with AG and CagA+ *H. pylori* infection are at high risk for gastric cancer and screening programs should focus on the identification and treatment of these two groups. Further studies are needed to evaluate the feasibility, potential for adverse conditions and cost-effectiveness of such a targeted screening and eradication program in Western populations with low to intermediate gastric cancer risk.

## MATERIALS AND METHODS

### Study design

The ESTHER study (*Epidemiologische Studie zu Chancen der Verhütung, Früherkennung und optimierten Therapie chronischer Erkrankungen in der älteren Bevölkerung* [German]) is a population-based prospective cohort study conducted in Saarland, a federal state in the southwest of Germany. A total of 9,949 participants aged 50–75 years were recruited by their general practitioners during a general health check-up between July 2000 and December 2002. Those with insufficient knowledge of the German language were excluded. Details of the study design have been reported elsewhere [44–46].

### Ethics

The study was approved by the ethics committees of the medical faculty of the University of Heidelberg and of the medical board of the German federal state of Saarland. Written informed consent was obtained from each participant.

### Data collection

### Questionnaires

A standardized questionnaire was used to collect comprehensive information on participants’ sociodemographic and lifestyle characteristics at baseline. General practitioners completed a standardized health check-up form and provided medical information.

### Serologic examinations

Serum samples were obtained from all participants at baseline and stored at −80°C until analysis. All samples were analyzed by enzyme-linked immunosorbent assay (ELISA) for the presence of immunoglobulin G (IgG) antibodies both against *H. pylori* in general and specific to the CagA of *H. pylori* (*H. pylori* Screening ELISA and *H. pylori* p120 [CagA] ELISA; ravo *H. Pylori* Diagnostika, Freiburg, Germany). Classification of infection status was performed according to the manufacturer's instructions and borderline results were considered negative. Additionally, serum concentrations of pepsinogen (PG) I and II were determined by ELISA (Biohit, Helsinki, Finland).

Participants were categorized based on their serostatus of *H. pylori* IgG and CagA, as: (i) non-infected (IgG–/CagA–), (ii) carrying CagA– *H. pylori* infection (IgG+/CagA–), and (iii) carrying CagA+ *H. pylori* infection (IgG+/CagA+). For presence of AG, the following commonly employed serological classification was used: PG I < 70 ng/mL and PG I/II ratio < 3.0 [41, 47, 48].

### Follow-up

Follow-up with respect to cancer incidence and mortality was conducted by record linkage with the statewide Saarland Cancer Registry through December 31st, 2011. The completeness of the cancer registry data was further increased by linkage with mortality statistics of the Saarland with collected death certificates of all deceased inhabitants and collecting information on newly developed cancers by questionnaires to the study participants at the 2-, 5- and 8-year follow-up of the ESTHER study (response rates among survivors: 96%, 88% and 79%, respectively). Self-reported cancers were validated by inquiry at the general practitioners of the study participants and only confirmed cases were considered as cases. Cancers specifically addressed in this analysis include gastric cancer (International Classification of Diseases 10th version [ICD-10] code: C16), colonic cancer (C18) and pancreatic cancer (C25). For gastric cancer, an additional distinction was made between cardia cancer (C16.0) and non-cardia gastric cancer (all except C16.0 in C16 classification).

### Population

**Figure 3:**
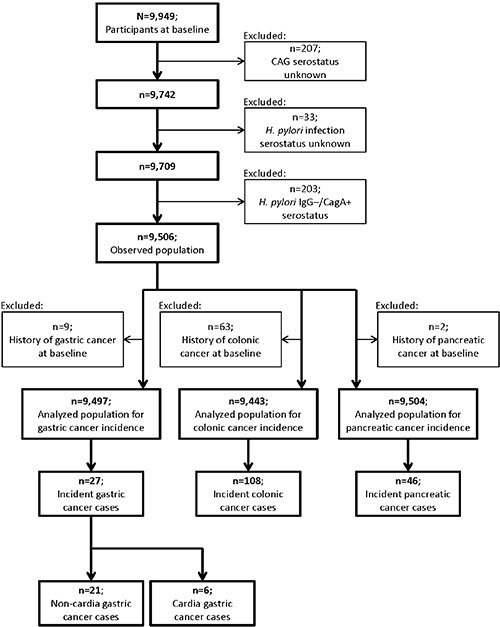
Flow chart for selection of the study population

Figure 3 shows the flow chart for the selection of the study population. Participants without measurement of AG or *H. pylori* infection were excluded. In order to minimize potential misclassification of infection status, we also excluded 203 participants with IgG–/CagA+ *H. pylori* serostatus, which might often reflect past infection [49]. Among 9,056 remaining participants, 9, 63 and 2 subjects had a history of gastric, colonic and pancreatic cancer before baseline, respectively, and were excluded from analyses of the incidence of the respective cancer.

### Statistical analysis

General characteristics of the population by *H. pylori* status were compared by Chi-square test for categorical variables and Wilcoxon test for ranked variables. Univariate time-to-event analyses were performed using Kaplan-Meier curves and log-rank tests were applied to compare incidence among the following groups of participants: non-infected, all infected, infected with non-virulent or with virulent strains. In addition, multivariate analyses were performed by Cox proportional hazards model after checking the proportional hazards assumption by the Kolmogorov-type supremum test based on a sample of 1,000 Martingale residual patterns (no statistically significant violations observed; data not shown), and hazard ratios (HRs) with 95% confidence intervals (CIs) were estimated. Cox models were adjusted for age, gender, education level, smoking and alcohol consumption. Cox regression was also used to separately compare the incidence of gastric, colonic and pancreatic cancer between non-atrophic and atrophy-presenting subjects, respectively. Additionally, in analyses for all gastric cancer or non-cardia cancer only, the non-atrophic and atrophy-presenting persons were further stratified by *H. pylori* serostatus. All statistical tests were 2-sided and statistical significance was defined as *p* < 0.05. SAS, version 9.2, statistical software (SAS Institute, Cary, North Carolina) was used for statistical analyses.

Multiple imputation was employed to impute missing values in baseline covariates. Information on age and sex was complete and the proportions of missing values for education, smoking and alcohol consumption were 2.5%, 2.8% and 9.7%, respectively. To the best of our knowledge, data were missing at random, which was the assumption of the multiple imputation. The variables age, sex, education level, smoking and alcohol consumption were used for the imputation model and 20 data sets were imputed with the SAS 9.2 procedure “PROC MI”, using the *Markov chain Monte Carlo* method. All multivariable analyses were performed in the 20 imputed data sets and results of the individual data sets were combined by the SAS 9.2 procedure “PROC MIANALYZE”, taking the variation between the results of the imputed data sets into account.
